# Intra-Articular Cyclo(His-Pro) Attenuates Monosodium Iodoacetate-Induced Osteoarthritis by Suppressing COX-2/PGE_2_ Signaling and Cartilage Catabolism in Rats

**DOI:** 10.3390/ijms27114742

**Published:** 2026-05-25

**Authors:** Gyuwon Huh, Dohyun Lee, Jongsu Jeon, Daehun Kim, Hoe-Yune Jung

**Affiliations:** 1 R&D Center, NovMetaPharma Co., Ltd., Pohang 37668, Republic of Korea; gwhuh@novmeta.com (G.H.);; 2 Division of Interdisciplinary Bioscience and Bioengineering, Pohang University of Science and Technology (POSTECH), Pohang 37673, Republic of Korea

**Keywords:** osteoarthritis, Cyclo(His-Pro), monosodium iodoacetate, intra-articular injection, COX-2/PGE_2_, cartilage catabolism

## Abstract

Osteoarthritis (OA) remains an alarming therapeutic challenge, as conventional intra-articular interventions primarily address symptomatic relief without halting progressive cartilage and bone degeneration. In this study, we investigated the disease-modifying potential of Cyclo(His-Pro) (CHP) in a monosodium iodoacetate (MIA)-induced OA rat model. Intra-articular CHP yielded significant clinical improvements, reducing joint edema and reversing OA-induced mechanical and thermal hypersensitivity, as evidenced by lifting behavior, rotarod performance, and hot plate tests. Beyond analgesia, micro-computed tomography (micro-CT) analysis showed that CHP preserved subchondral bone architecture, restoring trabecular volume and thickness and reducing serum C-terminal telopeptide of type II collagen (CTX-2), indicative of suppressed cartilage degradation. At the molecular level, CHP reprogrammed the joint microenvironment by suppressing *Cox2*, *Adamts5*, *Mmp13*, *Mmp1*, *Mmp2*, and *Timp2* expression and decreasing systemic prostaglandin E2 (PGE_2_) levels. Moreover, CHP showed efficacy comparable to Conjuran, a polynucleotide-based mechanical supportive agent, while additionally targeting COX-2/PGE_2_-driven inflammatory cascades and cartilage catabolic pathways. Collectively, these findings indicate that intra-articular CHP confers combined analgesic, chondroprotective, and osteoprotective effects, supporting its potential as a promising disease-modifying osteoarthritis drug candidate.

## 1. Introduction

Osteoarthritis (OA) is a disorder that weakens joints and affects millions of people globally, characterized by joint pain, stiffness, and structural changes [1,2]. The pathology of OA involves the entire joint organ, including articular cartilage, subchondral bone, synovium, and surrounding muscles [2,3]. A critical aspect of OA progression is the chronic inflammatory process and subsequent catabolic effects that lead to the irreversible breakdown of articular cartilage [4,5]. In Northeast Asia, the trajectory of OA incidence is particularly alarming [6,7]. South Korea, for example, exhibits one of the highest age-standardized prevalence rates globally, with specific reports identifying it as a leading nation in knee OA burden [8,9]. This rapidly increasing burden in East Asia, particularly in South Korea, underscores the urgent need for locally relevant, effective, and safe intra-articular interventions that can modify structural disease progression beyond transient symptomatic relief. Moreover, recent evidence emphasizes that OA is a multifaceted disease driven by a complex interplay of demographic aging, sedentary lifestyles, and altered mechanobiology. These upstream etiological factors—ranging from chronic underloading to excessive mechanical stress—converge on key molecular pathways, such as the activation of catabolic mediators (e.g., ADAMTS5 and MMPs) and the COX-2/PGE_2_ signaling axis, which collectively disrupt cartilage homeostasis [10].

Current therapeutic options for OA are primarily analgesics, non-steroidal anti-inflammatory drugs (NSAIDs), and corticosteroids, which are associated with various adverse effects and fail to arrest disease progression [11,12,13]. While intra-articular injectables such as hyaluronic acid (HA) and polynucleotides (PN) have been developed to provide mechanical and supportive benefits, these interventions often show limited structural efficacy and may present potential iatrogenic risks associated with the procedure itself [14,15,16]. Consequently, there remains a critical unmet need for effective disease-modifying OA drugs (DMOADs) that can target the underlying pathophysiology.

However, these agents predominantly act through visco-supplementation or mechanical cushioning of the joint space, with limited ability to directly suppress inflammatory cascades or catabolic mediators that drive irreversible cartilage and subchondral bone damage. Consequently, there remains a critical gap for intra-articular therapeutics that can simultaneously alleviate pain and actively modulate the molecular drivers of structural joint degeneration. This highlights the unmet need for disease-modifying osteoarthritis drugs (DMOADs).

Cyclo(His-Pro) (CHP) is an endogenous cyclic dipeptide that has been reported to exert anti-inflammatory and cytoprotective effects by modulating NF-κB and Nrf2 signaling and suppression of NLRP3 inflammasome activation [17,18,19]. Persistent activation of NF-κB, oxidative stress, and inflammasome signaling are key upstream drivers of COX-2 induction, prostaglandin E2 (PGE_2_) overproduction, and subsequent upregulation of cartilage-degrading enzymes in OA. These pleiotropic actions of CHP on inflammatory and oxidative pathways, therefore, provide a strong mechanistic rationale for exploring its potential as a disease-modifying candidate in knee OA [17,18,20]. Moreover, upstream biomechanical stressors and sedentary lifestyle-induced metabolic alterations are known to disrupt cartilage homeostasis, triggering a cascade of downstream inflammatory responses [21]. These upstream determinants converge on key signaling pathways, such as the COX-2/PGE_2_ axis, which further accelerates cartilage degradation. In this context, while OA management requires a multimodal approach, our study specifically focuses on evaluating how CHP modulates these converged downstream pathways to exert its chondroprotective effects. This rationale is further supported by our previous studies showing that CHP can modulate inflammatory and oxidative stress-related pathways *in vivo* [22,23,24].

The monosodium iodoacetate (MIA)-induced OA model in rodents is a widely accepted preclinical model that mimics the structural and functional changes observed in human OA, including inflammation, pain, and cartilage degradation [25,26,27,28,29]. Moreover, this model provides a reliable platform for evaluating potential therapeutic agents targeting both symptomatic and structural aspects of the disease [25,26,28].

Therefore, this study aimed to evaluate the disease-modifying potential of intra-articular CHP in a well-established MIA-induced rat model. We hypothesized that CHP would attenuate joint inflammation, restore COX-2/PGE_2_-driven catabolic signaling homeostasis, and preserve cartilage and subchondral bone structure, thereby improving pain-related functional outcomes. In addition, we compared the therapeutic effects of CHP with those of Conjuran, a clinically used polynucleotide-based intra-articular agent, to position CHP relative to an established mechanical supportive treatment.

## 2. Results

### 2.1. CHP Reduces Knee Edema and Improves Behavioral Outcomes

**Figure 1:**
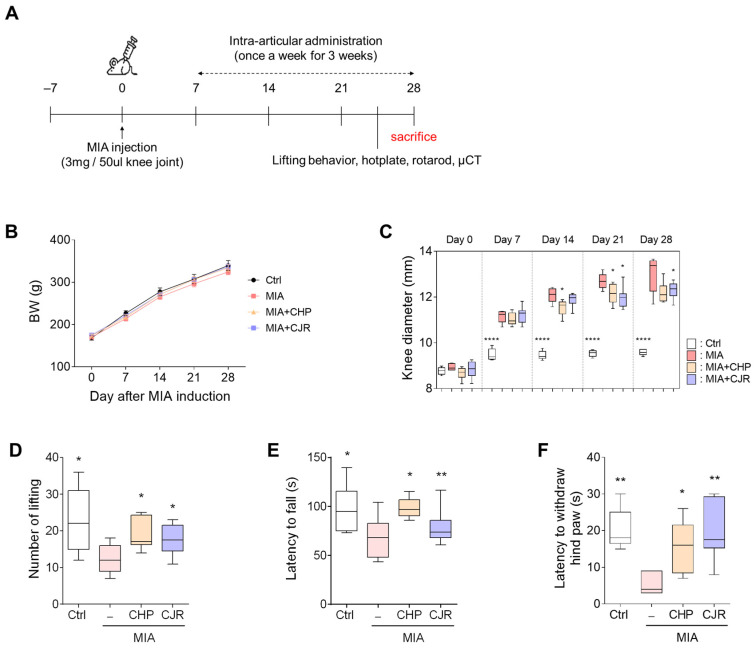
Effects of CHP on the MIA-induced OA rat model. (**A**) Scheme of the *in vivo* schedule for the MIA-induced OA rat model. (**B**) No difference in body weight (BW) was observed between MIA-induced OA rats and drug-administered groups. Data are shown as mean ± SEM. (**C**) CHP significantly reduced knee swelling in MIA-induced OA rats. (**D**,**E**) CHP improved mobility in the lifting behavior test and rotarod test. (**F**) CHP restored pain sensitivity to near-normal levels in the hot plate test. Data are presented as box-and-whisker plots (median, IQR, and min-to-max whiskers). Significant differences were assessed by Student’s *t*-test (* *p* < 0.05, ** *p* < 0.01, and **** *p* < 0.0001 versus MIA group).

Throughout the experimental period (Figure 1A), all groups maintained consistent body weight trends with no signs of treatment-related toxicity (Figure 1B). MIA-induced osteoarthritis resulted in significant knee joint edema and pain, both of which were significantly mitigated by CHP treatment, with efficacy comparable to Conjuran (Figure 1C). On day 14, one week after drug administration, the MIA group showed a significant increase in knee diameter (12.09 ± 0.14 mm) compared with that of the control group (9.46 ± 0.09 mm). In contrast, CHP or Conjuran significantly reduced knee diameter (11.54 ± 0.14, 11.89 ± 0.10 mm, respectively; Figure 1C).

To evaluate pain and OA-associated functional impairment, a series of behavioral tests was conducted. In the lifting behavior test, the number of lifting in the MIA group showed a significant reduction (12.40 ± 1.81 times) compared with the control group (22.80 ± 4.03 times; Figure 1D), suggesting impaired knee bending due to joint swelling and pain. In contrast, CHP treatment significantly improved lifting behavior compared with the MIA group (19.00 ± 1.81 times; Figure 1D), indicating recovery of joint function and pain-related functional impairment. Consistently, in the rotarod test, the MIA group showed a significant reduction in endurance (68.25 ± 7.18 s) compared with the control group (97.19 ± 8.26 s). In contrast, CHP treatment significantly restored endurance compared with the MIA group (98.81 ± 3.46 s; Figure 1E). In the hot plate test, the withdrawal latency of the right hind paw was significantly decreased in the MIA group (5.60 ± 1.40 s) compared with the control group (20.20 ± 2.58 s; Figure 1F), indicating increased pain sensitivity. In contrast, CHP administration significantly increased withdrawal latency (15.67 ± 2.87 s; Figure 1F), suggesting attenuation of pain responses.

### 2.2. CHP Protects Subchondral Bone Structure and Reduces Cartilage Turnover

**Figure 2:**
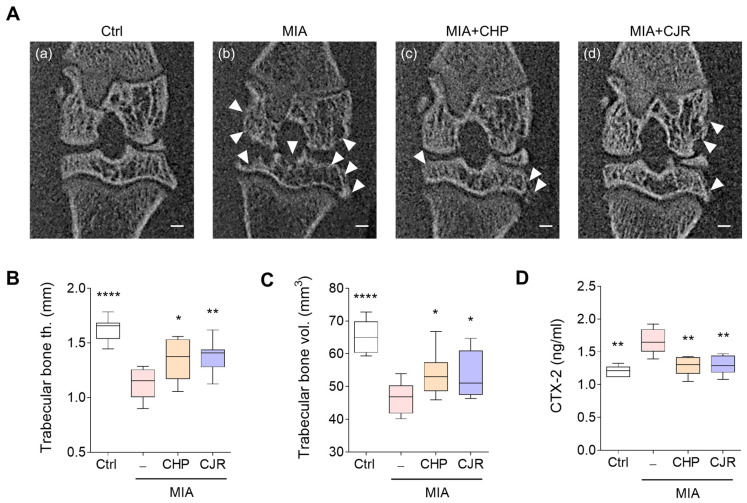
Effects of CHP on the MIA-induced OA rat model. (**A**) Representative micro-CT images of the knee joint. The images were acquired at a voxel size (spatial resolution) of 9 µm. Scale bar = 1 mm. White arrows indicate tidemark of damaged trabecular bone structure on day 24 after the MIA injection. (**a**) Control (Ctrl, *n* = 5); (**b**) MIA (*n* = 5); (**c**) MIA + CHP (*n* = 6); (**d**) MIA + CJR (*n* = 8). Osteoarthritis was induced by intra-articular injection of MIA. CHP or Conjuran was administered after MIA injection. (**B**) Quantitative analysis of trabecular bone thickness. (**C**) Quantitative analysis of trabecular bone volume. (**D**) Serum CTX-2 levels indicated that CHP protected against cartilage degradation in the MIA-induced OA rats. Data are presented as box-and-whisker plots (median, IQR, and min-to-max whiskers). Significant differences were assessed by Student’s *t*-test (* *p* < 0.05, ** *p* < 0.01, and **** *p* < 0.0001 versus MIA group).

Alterations in subchondral bone microarchitecture serve as a definitive structural hallmark, providing a direct and measurable assessment of joint-induced osteological compromise [30]. Micro-computed tomography (micro-CT) analysis revealed that MIA significantly deteriorated subchondral trabecular bone, as evidenced by marked reductions in both trabecular thickness (Tb.Th: 1.13 ± 0.05 mm) and bone volume fraction (BV/TV: 46.44 ± 1.70%) compared to the control group (Tb.Th: 1.63 ± 0.04 mm; BV/TV: 65.10 ± 1.74%; Figure 2B,C). Notably, CHP treatment significantly attenuated these structural changes, partially restoring trabecular integrity (Tb.Th: 1.35 ± 0.07 mm; BV/TV: 53.70 ± 2.35%). Consistently, serum levels of the cartilage degradation marker C-terminal telopeptide of type II collagen (CTX-2), were significantly elevated in the MIA group (1.67 ± 0.09 ng/mL) compared with the control group (1.20 ± 0.04 ng/mL; Figure 2D), whereas CHP treatment significantly reduced CTX-2 levels (1.28 ± 0.06 ng/mL), supporting its protective effects against joint degeneration.

### 2.3. CHP Suppresses Inflammatory and Catabolic Marker Expression

**Figure 3:**
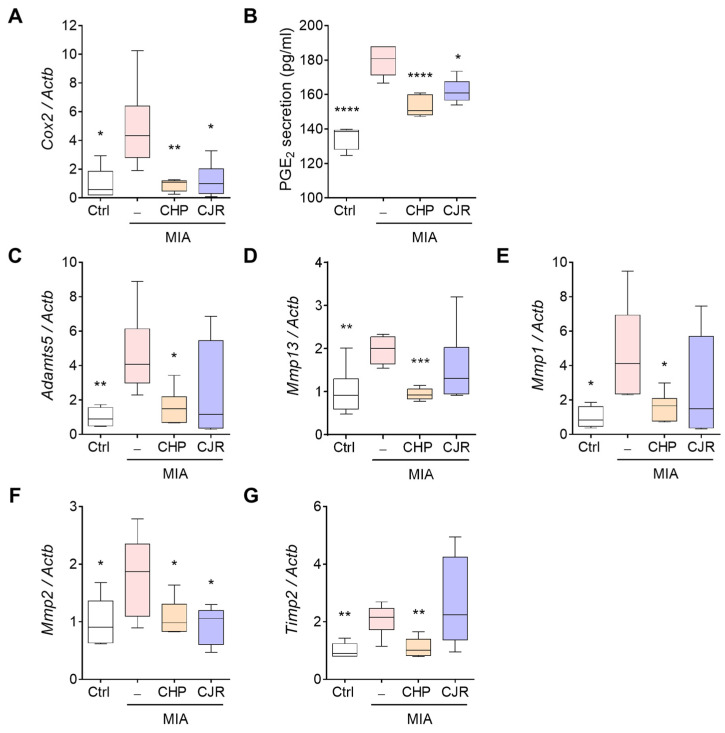
Effects of CHP on the MIA-induced OA rat model. (**A**) *Cox2* expression normalized to *Actb*. (**B**) Serum PGE_2_ levels indicated that CHP attenuated inflammatory signaling in MIA-induced OA rats. (**C**–**G**) *Adamts5*, *Mmp13*, *Mmp1*, *Mmp2*, and *Timp2* expression normalized to *Actb*. Data are presented as box-and-whisker plots (median, IQR, and min-to-max whiskers). Significant differences were assessed by Student’s *t*-test (* *p* < 0.05, ** *p* < 0.01, and *** *p* < 0.001, and **** *p* < 0.0001 versus MIA group).

To investigate the molecular effects of CHP in cartilage tissues, we analyzed transcriptional expression of key inflammatory and catabolic markers. MIA injection significantly increased the expression of cyclooxygenase-2 (*Cox2*), a critical enzyme responsible for prostaglandin synthesis, and its downstream mediator PGE_2_, which contributes to the amplification of pain and inflammation (*Cox2*: 4.84 ± 1.18 fold; PGE_2_: 179.43 ± 3.41 pg/mL; Figure 3A,B). In contrast, CHP treatment significantly downregulated *Cox2* expression and PGE_2_ levels (*Cox2*: 0.90 ± 0.17 fold; PGE_2_: 152.89 ± 2.38 pg/mL). In addition, MIA markedly increased cartilage degradation-related enzymes, including thrombospondin motifs 5 (*Adamts5*) and matrix metalloproteinases (MMPs) (*Adamts5*: 4.63 ± 0.95 fold; *Mmp13*: 1.97 ± 0.15 fold; *Mmp1*: 4.54 ± 1.31 fold; *Mmp2*: 1.80 ± 0.28 fold), as well as the matrix remodeling-related marker *Timp2* (2.08 ± 0.22 fold). CHP treatment significantly reduced the expression of these catabolic markers (*Adamts5*: 1.59 ± 0.41 fold; *Mmp13*: 0.94 ± 0.06 fold; *Mmp1*: 1.60 ± 0.34 fold; *Mmp2*: 1.08 ± 0.13 fold; *Timp2*: 1.10 ± 0.14 fold; Figure 3C–G). These data suggest that CHP significantly suppressed cartilage inflammation and catabolism.

## 3. Discussion

In this study, intra-articular administration of CHP significantly ameliorated MIA-induced knee OA by improving joint edema and pain-related behaviors, preserving subchondral bone microarchitecture, and reducing systemic cartilage degradation markers. At the molecular level, CHP suppressed COX-2 expression and PGE_2_ production and downregulated key catabolic enzymes, including ADAMTS5 and multiple MMPs, thereby reprogramming the inflammatory and degradative joint microenvironment. Notably, these therapeutic effects were comparable to those of Conjuran across functional and structural readouts, while CHP additionally exerted robust anti-inflammatory and anti-catabolic actions, supporting its disease-modifying potential.

OA progression is closely associated with inflammatory activation, particularly by oxidative stress and NF-κB pathway [31]. In the MIA-induced OA model, intra-articular MIA injection triggers a robust inflammatory cascade and progressive osteochondral damage by increasing reactive oxygen species (ROS) production and activating the NF-κB signaling pathway [31]. This leads to the upregulation of Cox2 and subsequently increases the production of PGE_2_ [32]. As PGE_2_ amplifies pain sensitization and inflammatory responses, secreted PGE_2_ acts in the cartilage microenvironment to further propagate inflammatory signaling and promote the expression of cartilage-degrading enzymes [33,34]. While PGE_2_ is known to play a dual role in cartilage biology—where physiological signaling can support homeostasis—its chronic overproduction under inflammatory conditions is a well-established driver of catabolic processes [35,36]. In this study, CHP treatment effectively attenuated the excessive PGE_2_ signaling and subsequent catabolic enzyme expression. Combined with the improved structural outcomes observed in micro-CT analysis, our results suggest that CHP does not merely inhibit PGE_2_ but rather restores a more favorable, balanced joint microenvironment by modulating upstream inflammatory cascades.

Consistent with this, our results showed that MIA significantly increased *Cox2* expression and PGE_2_ levels, accompanied by upregulation of key catabolic mediators, including *Adamts5* and matrix metalloproteinases (MMPs). These enzymes are known to break down the extracellular matrix, leading to progressive cartilage degradation [37,38,39]. In addition, the concurrent increase in *Timp2* suggests a dysregulated matrix remodeling environment, indicating altered extracellular matrix homeostasis in the MIA model [37,38,39]. Importantly, CHP treatment significantly suppressed *Cox2* expression and PGE_2_ production, along with downregulation of *Adamts5* and MMPs, indicating that CHP effectively interrupts these inflammation and catabolism pathways in OA progression. Given that CHP has been shown to modulate NF-κB and inflammasome activation in other inflammatory settings, the observed restoration of COX-2 and PGE_2_ balance in this OA model suggests that CHP may act upstream of this axis to dampen both nociceptive and structural components of disease progression.

Furthermore, MIA-induced mechanical and inflammatory stress activates *Adamts5*, a key aggrecanase responsible for proteoglycan degradation, leading to disruption of cartilage matrix integrity [40,41,42]. This structural collapse is reflected by increased levels of CTX-2, a well-established biomarker of type II collagen breakdown and subchondral bone remodeling [43,44]. Consistent with this, CHP treatment reduced CTX-2 levels and preserved subchondral bone architecture, indicating protection against structural joint damage. The concomitant reduction in CTX-2 levels and preservation of subchondral trabecular thickness and bone volume fraction further indicate that CHP modulates bone–cartilage crosstalk rather than acting solely on cartilage. Given that pathological remodeling at the osteochondral junction amplifies mechanical stress and propagates inflammatory signaling across the joint, simultaneous protection of cartilage and subchondral bone is considered a key attribute of potential DMOADs. These findings suggest that anti-catabolic effects of CHP extend beyond molecular regulation to the preservation of joint structure, supporting its potential as a DMOAD.

The therapeutic efficacy of CHP was broadly comparable to that of Conjuran across behavioral tests, micro-CT parameters, and CTX-2 levels in this MIA-induced OA model. While Conjuran is primarily regarded as a polynucleotide-based visco-supplement that provides mechanical cushioning and supportive effects within the joint space, CHP additionally exerted marked suppression of COX-2/PGE_2_ signaling and multiple catabolic enzymes, highlighting a mechanistically distinct profile.

In addition to its therapeutic efficacy, the safety profile of CHP further supports its translational potential. A previous clinical study in healthy subjects reported that oral administration of high-dose CHP plus zinc was well tolerated without significant adverse effects, suggesting a favorable systemic safety margin [45]. Although intra-articular administration may confer an even more favorable risk–benefit balance due to localized exposure, comprehensive evaluation of local tolerability, immunogenicity, and long-term safety in chronic OA settings will be essential in future studies. From a translational perspective, the robust efficacy observed after once-weekly intra-articular dosing in rats provides a rationale to explore clinically feasible dosing intervals and potential combination strategies with existing visco-supplements or analgesics.

This study has several limitations that should be acknowledged. First, only a single dose and dosing schedule of CHP were evaluated in one chemically induced OA model, which may not fully capture dose–response relationships or efficacy in post-traumatic or spontaneous OA models. Second, although gene expression of inflammatory and catabolic mediators was assessed in whole joint tissues, cell type-specific mechanisms in chondrocytes, synoviocytes, and subchondral osteoclasts remain to be elucidated. Future studies using additional OA models, dose-ranging designs, and cell-specific mechanistic approaches, including NF-κB and inflammasome pathway analyses, will be important to further define the therapeutic window and molecular targets of CHP. Third, histological evaluation was not performed, limiting direct microscopic assessment of cartilage matrix integrity and chondrocyte morphology. Nevertheless, quantitative micro-CT analysis provided three-dimensional structural information on subchondral bone architecture and joint integrity, offering translationally relevant evidence of OA-associated structural changes in this model.

Collectively, these findings demonstrate that intra-articular CHP attenuates OA progression by suppressing COX-2/PGE_2_-driven inflammation, downregulating key catabolic enzymes, and preserving cartilage and subchondral bone structure. CHP therefore fulfills key features of a disease-modifying osteoarthritis candidate and may represent a promising next-generation intra-articular therapeutic strategy for OA that warrants further preclinical and clinical investigation.

## 4. Materials and Methods

### 4.1. Animal Model and Experimental Design

Five-week-old male Sprague-Dawley (SD) rats were purchased from Koatech (Pyeongtaek, Republic of Korea). All animals were housed in group cages at 23 ± 3 °C under a 12 h light/dark cycle and acclimated for one week. Rats had access to distilled water and a laboratory chow diet *ad libitum*. OA was induced by a single intra-articular injection of MIA (3 mg/50 μL) into the right knee joint using a 31-gauge needle (Figure 1A). One week after MIA injection, which corresponds to the early-stage of OA development characterized by initial chondrocyte damage and inflammatory response in this model, rats were randomly assigned to different treatment groups: Control (Ctrl: *n* = 5), MIA (*n* = 5), 50 mg/kg of CHP (MIA + CHP: *n* = 6), and 4.1 mg/kg Conjuran (MIA + CJR: *n* = 8). Treatment was administered via intra-articular injection once a week for three weeks to evaluate the therapeutic effects of CHP on OA progression. The animals were monitored weekly, and body weight and knee diameter were recorded before drug administration. Before each intra-articular injection, the right knees of rats were sprayed with 70% ethanol to avoid contamination.

### 4.2. Evaluation of Knee Edema and Behavioral Tests

As illustrated in Figure 1A, the anteroposterior knee diameter was measured weekly using a digital caliper starting from the date of MIA injection (Week 0). The measurements were recorded on day zero (the date of MIA injection) and every week after MIA injection until the end of the experiment. The mean knee diameter was then calculated for each group. Behavioral assessments were conducted to evaluate pain-related impairments during weeks 3–4. The lifting behavior test was performed to assess articular pain by counting spontaneous limb lifts or preference during 5 min of free movement in a 40 × 25 × 17 cm cage. A single lifting event (rearing behavior) was scored whenever the eyes reached above the top edge of the cage during a 5-min session. This behavioral assay provided a quantitative measure of the animal’s ability to maintain upright posture for vertical exploration under osteoarthritic conditions. The accelerating rotarod apparatus (Panlab, LE8500, Cornellà, Spain) was used to assess joint functional stability and motor coordination. Rats were placed on a rotating rod (4 rpm) and allowed a short acclimation period. Then, each rat underwent two trials with accelerating rotation from 4 to 40 rpm over 5 min (300 s). The latency to fall was recorded for each trial, and the mean value was calculated. Thermal nociceptive thresholds were evaluated using a modified hot plate assay (JD-A-10A; Jeungdo Bio & Plant, Seoul, Republic of Korea) [46]. Following a 15-min equilibration period at 50 °C, rats were placed on the heated surface to observe nocifensive behaviors, such as hind paw licking, lifting, or jumping. The latency to the first manifestation of these responses was recorded manually, with a rigorous 30-s cut-off imposed to preclude thermal tissue injury. Thermal hyperalgesia was quantified as a significant reduction in withdrawal latency compared to control benchmarks.

### 4.3. Micro-CT Analysis of Subchondral Bone (In Vivo)

Following the study period, *in vivo* micro-CT scans were performed to analyze the subchondral bone structure. Key parameters measured included Tb.Th and BV/TV. Micro-CT imaging was performed using an Inveon^®^ Multi-Modality micro-PET/SPECT/CT scanner (Siemens Preclinical Solutions, Knoxville, TN, USA) to evaluate the structural changes in the knee joint. Rats were anesthetized with 3% isoflurane and precisely positioned on the animal bed with the knee joint centered in the field of view. For the structural evaluation of the subchondral bone, the trabecular bone thickness was measured at the central region of the proximal tibial plateau from the reconstructed CT images. Additionally, the trabecular bone volume was calculated by defining a specific region of interest encompassing the trabecular compartment of the tibia. All image acquisition and quantitative analyses were conducted using the Inveon Acquisition Workplace software (Version 1.5).

### 4.4. Biochemical Marker Analysis

To quantify circulating inflammatory mediators and cartilage turnover markers, serum was isolated from whole blood obtained via cardiac puncture. Following a 30-min incubation at room temperature, samples were centrifuged at 3000 rpm for 10 min, with the resulting supernatant stored at −80 °C until analysis. Serum concentrations of prostaglandin E2 (PGE_2_) and C-terminal telopeptide of type II collagen (CTX-2) were determined using commercially available enzyme-linked immunosorbent assay (ELISA) kits (CUSABIO, Houston, TX, USA, and Elabscience, Houston, TX, USA, respectively) according to the manufacturers’ instructions. Absorbance was recorded at 450 nm, and protein levels were calculated from the respective standard curves. Undiluted aliquots were used to ensure maximum sensitivity.

### 4.5. Gene Expression Analysis (Real-Time Quantitative Polymerase Chain Reaction, RT-qPCR)

Total RNA was extracted from the knee joint tissues using NucleoZOL (Macherey-Nagel, Düren, Germany, MN740404.200). Then, 1 μg of total RNA was used for cDNA synthesis using the iScript cDNA synthesis kit (Bio-Rad, Richmond, CA, USA, BR1708891). Real-time qPCR was conducted using THUNDERBIRD™ Next SYBR™ qPCR Mix (Toyobo Co. Ltd., Osaka, Japan, QPX-201) to quantify mRNA expression levels of key inflammatory and catabolic genes: cyclooxygenase-2 (*Cox2*), matrix metalloproteinase-1 (*Mmp1*), matrix metalloproteinase-2 (*Mmp2*), matrix metalloproteinase-13 (*Mmp13*), Tissue inhibitor of metalloproteinase-2 (*Timp2*), and ADAM metallopeptidase with thrombospondin type 1 motif 5 (*Adamts5*). The protocol included an initial denaturation step at 95 °C for 1 min followed by 40 cycles of denaturation at 95 °C for 15 s, annealing at 60 °C for 15 s, and extension at 72 °C for 10 s. Primer pairs were designed using the Primer-BLAST tool (https://www.ncbi.nlm.nih.gov/tools/primer-blast/ (accessed on 19 May 2026)) provided by NCBI and the specificity was confirmed through melt curve analysis conducted by gradually heating samples from 65 °C to 95 °C in 0.5 °C increments, holding at each step for 10 s while monitoring fluorescence. The primer sequences are provided in Table 1. All primer sequences are listed in the 5′ to 3′ orientation. β-actin (*Actb*) was utilized as the internal reference gene (housekeeping gene) for normalizing target gene expression.

### 4.6. Statistical Analysis

Statistical analyses were performed using GraphPad Prism v.6.0. Data are presented as box-and-whisker plots indicating the median, interquartile range (IQR), and minimum-to-maximum whiskers. Grubbs’ test was applied a priori, and at most one outlier per group was excluded when *p* < 0.05. Differences between two independent groups were evaluated using an unpaired, two-tailed Student’s *t*-test under the assumption of homogeneity of variance, and *p*-values < 0.05 were considered statistically significant.

### 4.7. Generative AI Statement

**Figure 4:**
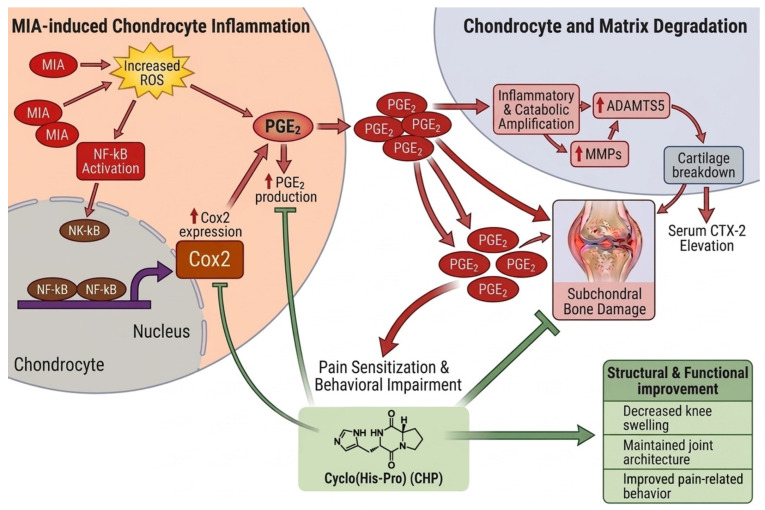
Schematic illustration of CHP-mediated attenuation of OA progression.

ChatGPT 5.0 (OpenAI) was used for language editing to improve the clarity and readability of the manuscript. Nano Banana 2 (Google) was used for graphical refinement of the schematic illustration (Figure 4). No generative AI tools were used for data generation, statistical analysis, or scientific interpretation. All data and conclusions were verified by the authors.

## Figures and Tables

**Table 1:** The sequence of primers used for qRT-PCR.

Gene Name	Species	Sequence (5′→3′)	Melting Temperature (*T_m_*)
*Cox2 F*	Rat	TGA CGA GCG ACT GTT CCA AA	58.9 °C
*Cox2 R*	Rat	AAA AGC AGC TCT GGG TCG AA	58.9 °C
*Mmp1 F*	Rat	TTG CTC ACA CAT TCC CAC CA	58.8 °C
*Mmp1 R*	Rat	TGT CAC TGT TGT CGG TCC AC	59.2 °C
*Mmp2 F*	Rat	TGG GGG AGA TTC TCA CTT TG	55.8 °C
*Mmp2 R*	Rat	CCA TCA GCG TTC CCA TAC TT	56.7 °C
*Mmp13 F*	Rat	CCT GTG ACT CTT GCG GGA AT	59.1 °C
*Mmp13 R*	Rat	TGC CAG TCA CAT CTA AGC CA	58.0 °C
*Timp2 F*	Rat	AGA TCA CAC GCT GCC CTA TG	58.9 °C
*Timp2 R*	Rat	TGG TGC CCA TTG ATG CTC TT	59.0 °C
*Adamts5 F*	Rat	GCA AAT CTT TCC GCC ACG AG	59.2 °C
*Adamts5 R*	Rat	GGA CAC CTG CGT ATT TGG GA	59.1 °C
*Actb F*	Rat	AGC AAG CAG GAG TAC GAT GAG	58.9 °C
*Actb R*	Rat	AAC GCA GCT CAG TAA CAG TCC	59.6 °C

## Data Availability

The original contributions presented in this study are included in the article. Further inquiries can be directed to the corresponding author.

## Abbreviations

BV/TV	bone volume/total tissue volume
CHP	Cyclo(His-Pro)
CJR	Conjuran
CTX-2	C-terminal telopeptide of type II collagen
DMOADs	disease-modifying osteoarthritis drugs
DW	distilled water
ELISA	Enzyme-linked immunosorbent assay
HA	hyaluronic acid
MIA	monosodium iodoacetate
micro-CT	Micro-computed tomography
NSAIDs	non-steroidal anti-inflammatory drugs
OA	Osteoarthritis
PGE_2_	prostaglandin E2
PN	polynucleotides
ROI	Region of interest
SD	Sprague-Dawley
SEM	standard error of the mean
Tb.Th	trabecular bone thickness
